# Functional analysis of a novel splice site variant in the ASAH1 gene

**DOI:** 10.1002/mgg3.2317

**Published:** 2023-11-14

**Authors:** Shujuan Yan, Fang Fu, Hang Zhou, Ruibin Huang, You Wang, Can Liao

**Affiliations:** ^1^ Prenatal Diagnostic Center, Guangzhou Women and Children's Medical Center Guangzhou Medical University Guangzhou Guangdong China

**Keywords:** acid ceramidase deficiency, ASAH1, Farber disease, hydrops fetalis, prenatal diagnosis, spinal muscular atrophy with progressive myoclonic epilepsy, whole exome sequence

## Abstract

**Background:**

Acid ceramidase (ACDase) deficiency is an ultrarare autosomal recessive lysosomal disorder caused by pathogenic N‐acylsphingosine amidohydrolase (*ASAH1*) variants. It presents with either Farber disease (FD) or spinal muscular atrophy with progressive myoclonic epilepsy (SMA‐PME).

**Objective:**

The study aims to identify a novel splice site variant in a hydrops fetus that causes *ASAH1*‐related disorder, aid genetic counseling, and accurate prenatal diagnosis.

**Methods:**

We report a case of hydrops fetalis with a novel homozygous mutation in *ASAH1* inherited from non‐consanguineous parents. We performed copy number variation sequencing (CNV‐Seq) and whole exome sequencing (WES) on the fetus and family, respectively. Minigene splicing analyses were conducted to confirm the pathogenic variants.

**Results:**

WES data revealed a splice site variant of the *ASAH1* (c.458‐2A>T), which was predicted to affect RNA splicing. Minigene splicing analyses found that the c.458‐2A>T variant abolished the canonical splicing of intron 6, thereby activating two cryptic splicing products (c.456_458ins56bp and c.458_503del).

**Conclusions:**

Overall, we identified a novel splice site variant in the mutational spectrum of *ASAH1* and its aberrant effect on splicing. These findings highlight the importance of ultrasonic manifestation and family history of fetal hydrops during *ASAH1*‐related disorders and could also aid genetic counseling and accurate prenatal diagnosis. To the best of our knowledge, this is the shortest‐lived account of *ASAH1*‐related disorders in utero with severe hydrops fetalis.

## Graphical abstract

We made an important discovery of a previously unidentified splice site variant within the extensive mutational landscape of *ASAH1* gene. This variant was observed in a hydrops fetus, adding to its clinical significance. Subsequently, we conducted a comprehensive and rigorous functional analysis to thoroughly examine the specific impact of this variant on splicing processes.

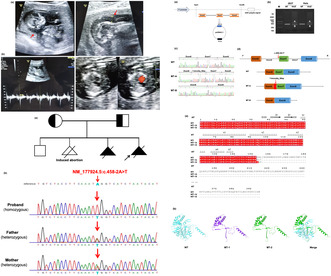

## 1 INTRODUCTION

Acid ceramidase deficiency is an ultrarare autosomal recessive lysosomal storage disorder caused by pathogenic variants of the *ASAH1*. Acid ceramidase (ACDase, EC 3.5.1.23) enzyme, which is usually responsible for the degradation of ceramide to sphingosine and free fatty acids within lysosomes, is ubiquitously expressed and localized in lysosomal and endosomal compartments. ACDase, encoded by *ASAH1*, is located on chromosome 8p21.3/22, which spans approximately 30 kb and comprises 14 exons and 13 introns. Its full‐length cDNA comprises a 1185 bp open reading frame corresponding to a primary translation product of 395 amino acids (Koch et al., 1996; Su et al., 2021).

Variations in lysosomal ACDase cause ceramides to accumulate inside lysosomes of various tissues. The most common phenotype of *ASAH1* defects, namely Farber lipogranulomatosis (OMIMI #228000), is characterized by typical symptoms, including early‐onset subcutaneous nodules, painful and progressively deformed joints, hoarseness by laryngeal involvement, as well as various combinations of respiratory, adult‐onset peripheral osteolysis, and central and peripheral nervous system manifestations. Spinal muscular atrophy with progressive myoclonic epilepsy (SMA‐PME, OMIM #159950), the less prevalent phenotype, is characterized by the childhood onset of proximal muscle weakness and muscular atrophy due to the degeneration of spinal motor neurons, followed by onset of myoclonic seizures. Disseminated lipogranulomatosis was first identified in a 14‐month‐old infant in 1947 and was later linked to pathogenic variants of the *ASAH1* in 1996 (Alayoubi et al., 2013; Farber, 1952). Numerous techniques have been developed for the diagnosis of FD. These include genetic testing to verify the pathogenic variants of *ASAH1* and/or measuring the activity of the ACDase enzyme in blood leukocytes or cultured fibroblasts. According to clinical reports, ACDase deficiency is extremely rare, with only about 200 FD patients reported in the literature so far (Burek et al., 2001; Yu et al., 2018). Most clinical phenotypes and disease progression have only been reported in children and adults. At present, the FD case with the shortest survival time is a female preterm infant at 29 weeks of gestation in 1996, who manifested severe hydrops fetalis and died 3 days after birth, and ceramide depositions in the spleen and cultured fibroblasts were observed (Schäfer et al., 1996).

Here, we present the case of a 13‐week‐old fetus from China, who presented with severe hydrops fetalis, abnormal cardiac structure, arrhythmia, and bradycardia. Genetic analysis revealed a novel homozygous mutation (c.458‐2A>T) in *ASAH1* that was inherited from non‐consanguineous parents. Minigene splicing analyses revealed that the c.458‐2A>T variant abolished the canonical splicing of intronic 6, thereby leading to the activation of two cryptic splicing products, namely a 56 bp insertion and exon 7 deletion, which caused either truncation or skipping of the downstream exon accompanied by a loss‐of‐function mechanism. Our findings highlight the importance of ultrasonic manifestation and family history of fetal hydrops during *ASAH1*‐related disorders.

## 2 MATERIALS AND METHODS

### 2.1 Samples

The index proband was a 14‐gestational‐week fetus with severe hydrops, which was the fourth pregnancy of the 29‐year‐old woman, who had a 9‐year‐old child, a previously induced termination of pregnancy due to nonmedical reasons, a history of fetal hydrops, and death in the uterus at 13 weeks of gestation, but without genetic tests (Figure 2a). The couple was non‐consanguineous and healthy. Results from a first‐trimester ultrasound, performed at 13 weeks of gestation, indicated that the index fetus had increased nuchal translucency (NT) thickness of 6.7 mm, severe hydrops fetalis, cardiac anomalies, arrhythmia, and bradycardia (65 beats/min). Despite the urgency of the current situation, the parents opted not to pursue invasive diagnostic testing to further analyze chromosome abnormalities. Upon their second visit to our center, it was evident that the fetus had died in utero at a gestational age of 14 weeks. The pregnancy was terminated. Macroscopic examination revealed moderate postmortem changes in a male fetus, with severe internal hydrops and two vessels in the umbilical cord. The parents did not consent to the autopsy and only allowed the collection of partial tissue for genetic analysis. DNA was extracted from the tissues and peripheral blood of the parents using the Qiagen DNA Blood Midi/Mini Kit (Qiagen GmbH, Hilden, Germany) according to the manufacturer's instructions.

### 2.2 Karyotype and copy number variation sequencing (CNV‐seq)

To determine the karyotype of the parents' peripheral blood, we used colchicine to arrest samples at metaphase via conventional karyotyping. Next, we performed G‐banding karyotyping at the 320–400 band level by analyzing 10 split phases. Briefly, genomic DNA was fragmented and libraries were constructed by end filling, adapter ligation, and PCR amplification. DNA libraries were subjected to CNV‐seq on a NextSeq 500 platform (Illumina, San Diego, CA, USA). Next, we uniquely and precisely mapped a total of 2.8–3.2 million reads onto the hg19 genomic sequence as a reference using the Burrows–Wheeler algorithm. Chromosome profiles were plotted as copy numbers (Y‐axis) relative to 20 kb count windows (*X*‐axis). Identified and mapped CNVs were queried against publicly available databases, including Decipher, Database of Genomic Variants (DGV), 1000 genomes, and Online Mendelian Inheritance in Man (OMIM), and pathogenicity was evaluated according to the guidelines outlined by the American College of Medical Genetics (ACMG) for the interpretation of sequence variants.

### 2.3 Whole exome sequencing

Whole exome sequencing (WES) of the DNA libraries was performed using the Hiseq XTen platform (Illumina, Inc., San Diego, CA, USA) to generate 150‐bp pair‐end reads. Raw reads were filtered and aligned to the human reference genome (hg38/GRCh38). The BAM files were generated by SNP analysis, duplication marking, indel realignment, and recalibration using GATK and SAMtools. The minor allele frequencies (MAFs) of all known variants were annotated according to the 1000 Genome Project, dbSNP EVS, ExAC, and gnomAD databases. Databases such as OMIM and ClinVar, multiple computational algorithms were used to predict the biological effects of candidates including SIFT, Human Splicing Finder, PolyPhen2, MutationTaster, PROVEAN, CADD, and MaxEntScan. Variant pathogenicity was evaluated based on standards and guidelines outlined by the American College of Medical Genetics and Genomics (ACMG) (Zhou et al., 2020).

### 2.4 Sanger sequencing

DNA was isolated via standard procedures and purified using a QIAamp DNA Blood Maxi Kit (Qiagen, Hilden, Germany) according to the manufacturer's instructions. The final variant was verified via Sanger sequencing using the following primers: F: GTCCCTCTTGTCTCAGCACTCA and R: GGTGAAGGCTTAGGATATTAGGATG. PCR was performed using standard methods. PCR products were then purified and sequenced on an ABI 3730 DNA Analyzer using BigDye Terminator v3.1 (Applied Biosystems).

### 2.5 Minigene analysis

The variant was subjected to in vitro minigene splicing assay as previously described (Richards et al., 2015). Summarily, wild‐type (WT) and mutant‐type (MT) forms of the minigene regions, encompassing exons 6–8 as well as introns 6 and 7 of *ASAH1*, were amplified via nested polymerase chain reaction (Nested PCR) from the proband's genomic DNA. The primer pairs used for amplification were 5′‐GAATGGAGTTTCTTGGCAGC‐3′ and 5′‐CAACCATGCGCTTTAAGGCC‐3′, 5′‐CCTGCCCAGATGTTCAGCT‐3′ and 5′‐AGATTGTGACTCCCAGCCCT‐3′. PCR products were cloned into a pcDNA3.1 reporter vector (Life Technologies, New York, USA) and double digested using KpnI and EcoRI enzymes. PCR and Sanger sequencing were used to verify the WT and MT expression plasmids prior to selection for subsequent transfection. Both 293T and HeLa cells were cultured and incubated for recombinant plasmid transient transfection with a Liposomal Transfection Reagent (40802ES03, YEASEN, Shanghai, China), as previously described (Richards et al., 2015). Next, total RNA was extracted from cells after 48 h using TRIzol reagent (Cowin Biotech Co., Jiangsu, China) according to the manufacturer's instructions. Reverse transcription‐PCR (RT‐PCR) was performed using the primer pair 5′‐CTAGAGAACCCACTGCTTAC‐3′ and 5′‐TAGAAGGCACAGTCGAGG‐3′. PCR fragments gene isoforms were determined via agarose gel electrophoresis and Sanger sequencing, respectively. Thereafter, the nucleotide sequence was translated into a protein sequence using the Expasy‐translate tool (https://web.expasy.org/translate/), followed by analysis of the effect of the mutation on translation.

## 3 RESULTS

### 3.1 Clinical features

**FIGURE 1:**
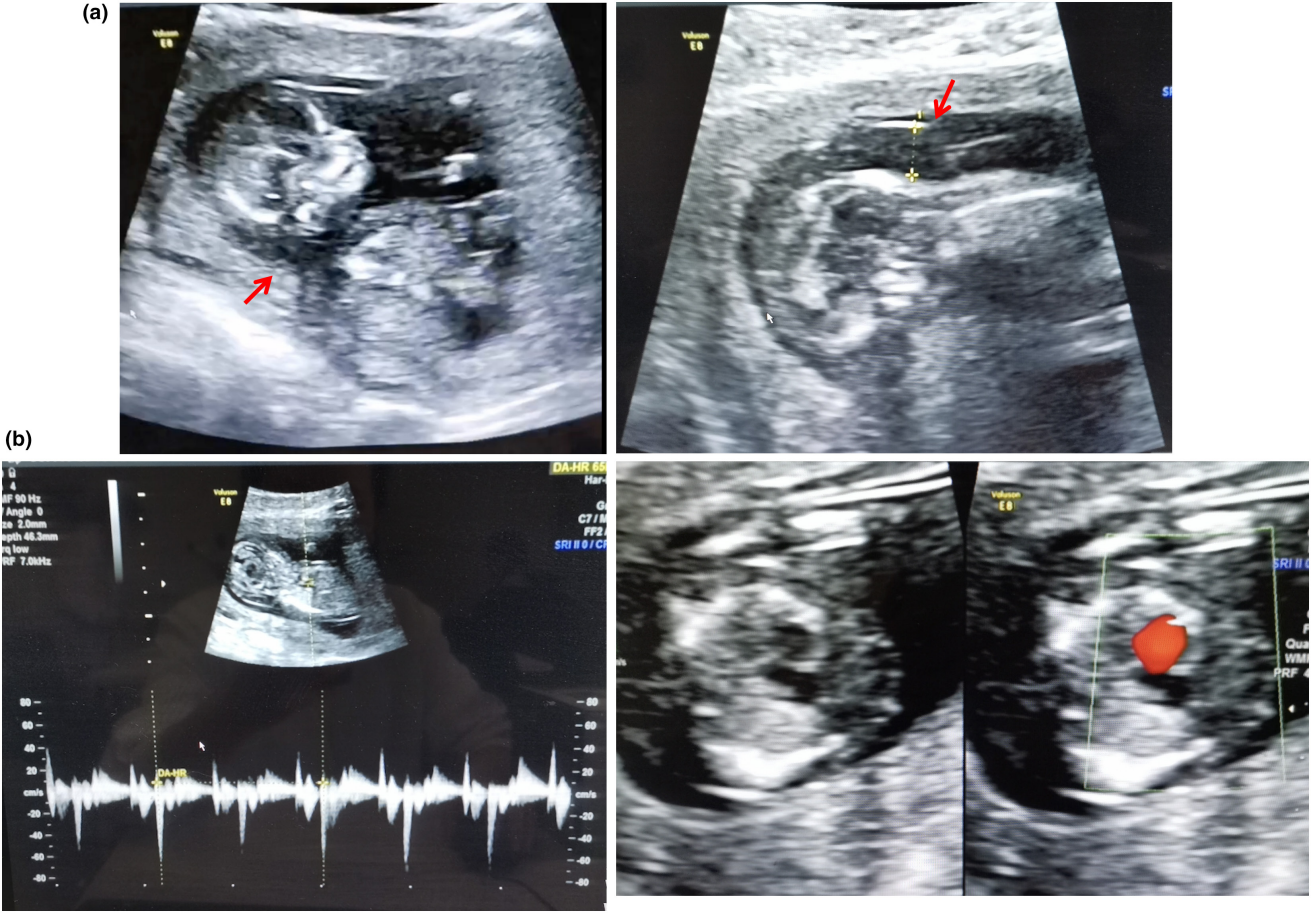
Clinical presentation of the fetus with *ASAH1*‐related disorder. (a) First trimester ultrasound showing increased NT thickness 6.7 mm (red arrow), severe hydrops fetalis, and cardiac anomalies. (b) Doppler ultrasound showed arrhythmia, bradycardia, and the ductus venosus alpha wave. The red arrow indicates the NT.

The first‐trimester ultrasound scan showed that a 13‐gestational‐week fetus had an increased NT thickness 6.7 mm, accompanied by severe hydrops fetalis, cardiac anomalies, arrhythmia, and bradycardia (65 beats/min). Doppler ultrasonography revealed a ductus venosus alpha wave (Figure 1).

### 3.2 Karyotype, CNV‐seq, and WES profiles

**FIGURE 2:**
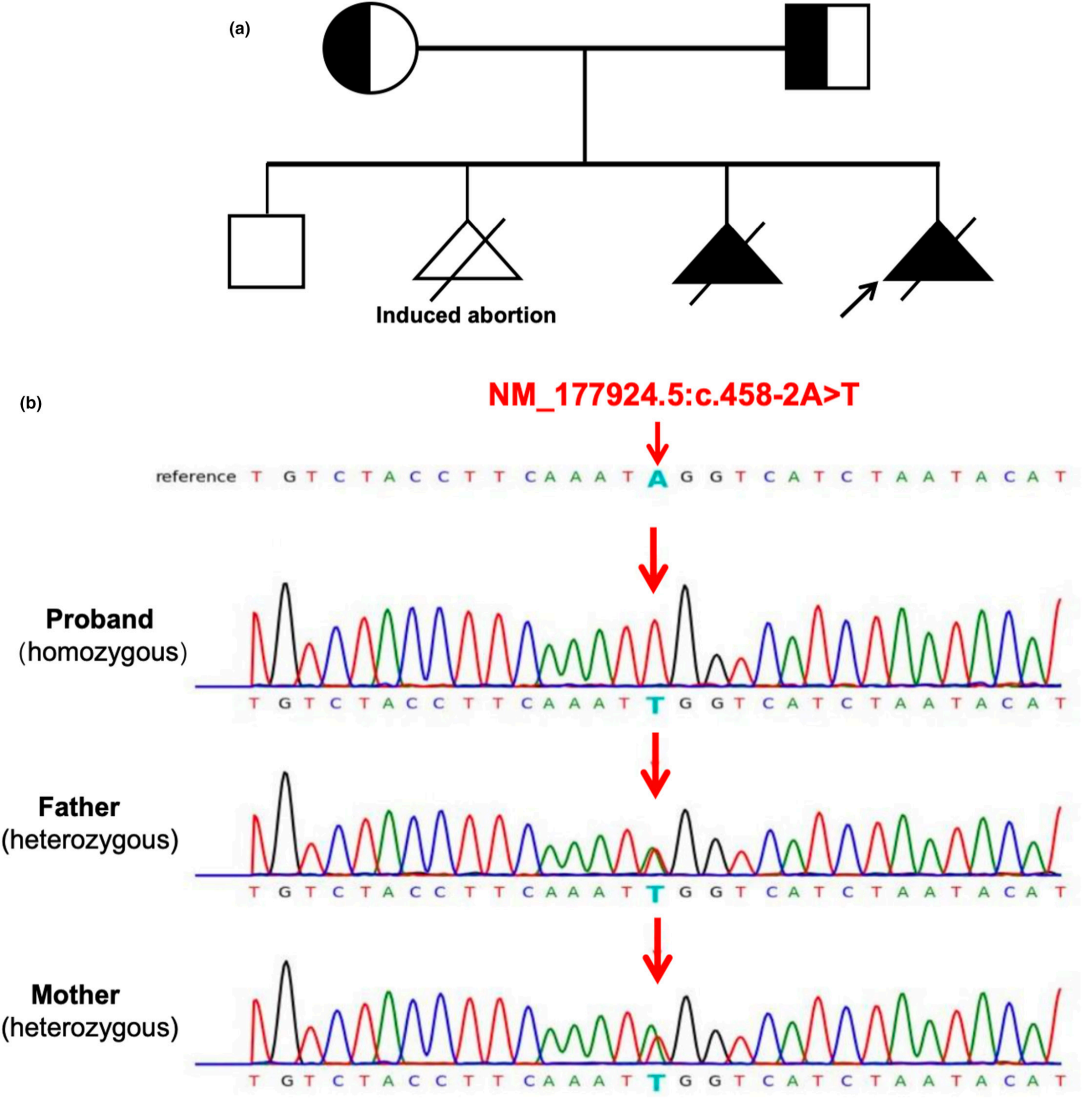
Pedigree and sequence analysis of the family. (a) The family pedigree. The black arrow indicates the proband. (b) The variant c.458‐2A>T of *ASAH1* was confirmed in the proband and non‐consanguineous parents. The red arrows indicate variant site.

Karyotype results in this couple, and the CNV‐seq profile of the fetus did not reveal any abnormalities. Consequently, the family consented, and we performed trio‐WES and identified a novel homozygous c.458‐2A>T variation in *ASAH1* (NM_177924.5) in the proband. However, both the unaffected parents were heterozygous. No other suspected variants were identified. The family pedigree is shown in Figure 2a. Sanger sequencing confirmed the presence of the *ASAH1* variant (Figure 2b). The c.458‐2A>T variant has never been reported in any public database (gnomAD, 1000Gennome, ExAC, EVS). Next, we employed SPIDEX, dbscSNV, spliceAI, NetGene2, and Beef Data and Genomics Programme databases to predict that the splice site variant (c.458‐2A>T) in *ASAH1* abrogated the intron 6 canonical splice site. ACMG guidelines suggest that it is likely to be pathogenic (PVS1 + PM2 + PM3_S) (Zhou et al., 2020).

### 3.3 The pathogenic *ASAH1* c.458‐2A>T gene variant

**FIGURE 3:**
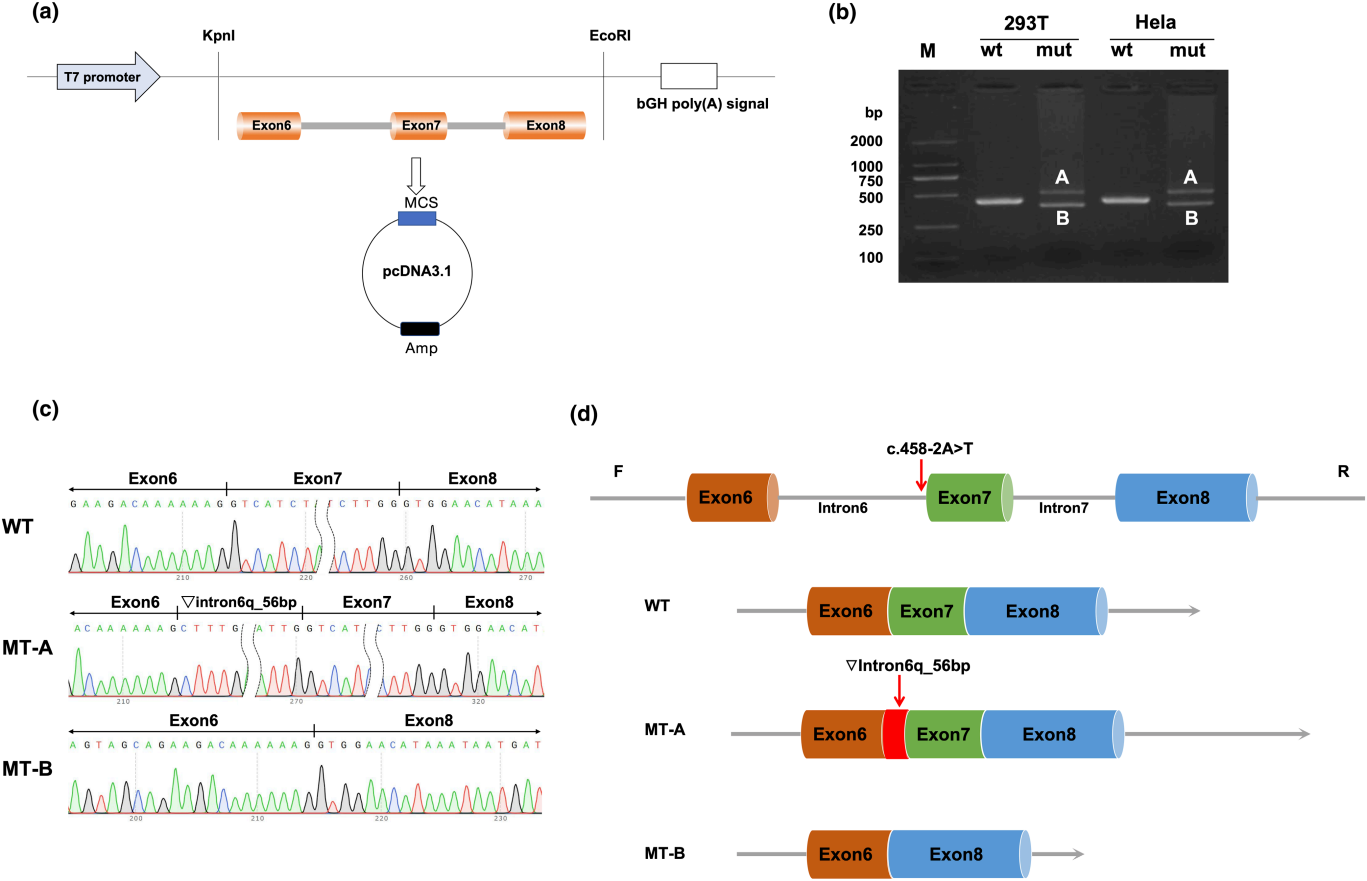
Minigene assay for *ASAH1* c.458‐2A>T variant and schematic representation of the splicing pattern. (a) The construction of minigene vector. (b) RT‐PCR results showing a single and two bands in the WT and MT, respectively (band a and b) in Hela and 293 T cell lines. (c) Minigene product sequencing demonstrated that the WT minigene formed normal mRNA, but the c.458‐2A>T substitution of *ASAH1* caused a splicing abnormality, which abrogate the intronic 6 canonical splice site and lead to activating two cryptic sites in exon 6, resulting in a 56 bp insertion (a) and exon 7 skipping (b); (d) Schematic representation of the splicing pattern in WT, MT‐A, and MT‐B.

A schematic representation of the minigene vector construct is shown in Figure 3a. RT‐PCR results revealed a single band for WT and two bands for MT both in HeLa and 293T cells (Figure 3b). Sanger sequencing results revealed normal splicing in the WT, but aberrant splicing of MT‐A and B, which resulted in the insertion of 56 nucleotides (lower band B) or skipping exon 7 (upper band A) (Figures 3c). The minigene splicing assay results suggested that the c.458‐2A>T substitution could abrogate the intron 6 canonical acceptor splice site and activate two cryptic sites in exon 6, which is predicted to cause a 56 bp insertion (c.456_458ins56bp p.Gly152Alafs*4) and exon 7 skipping (c.458_503del p.Gly153*4) (Figure 3d).

### 3.4 Bioinformatic analysis

**FIGURE 4:**
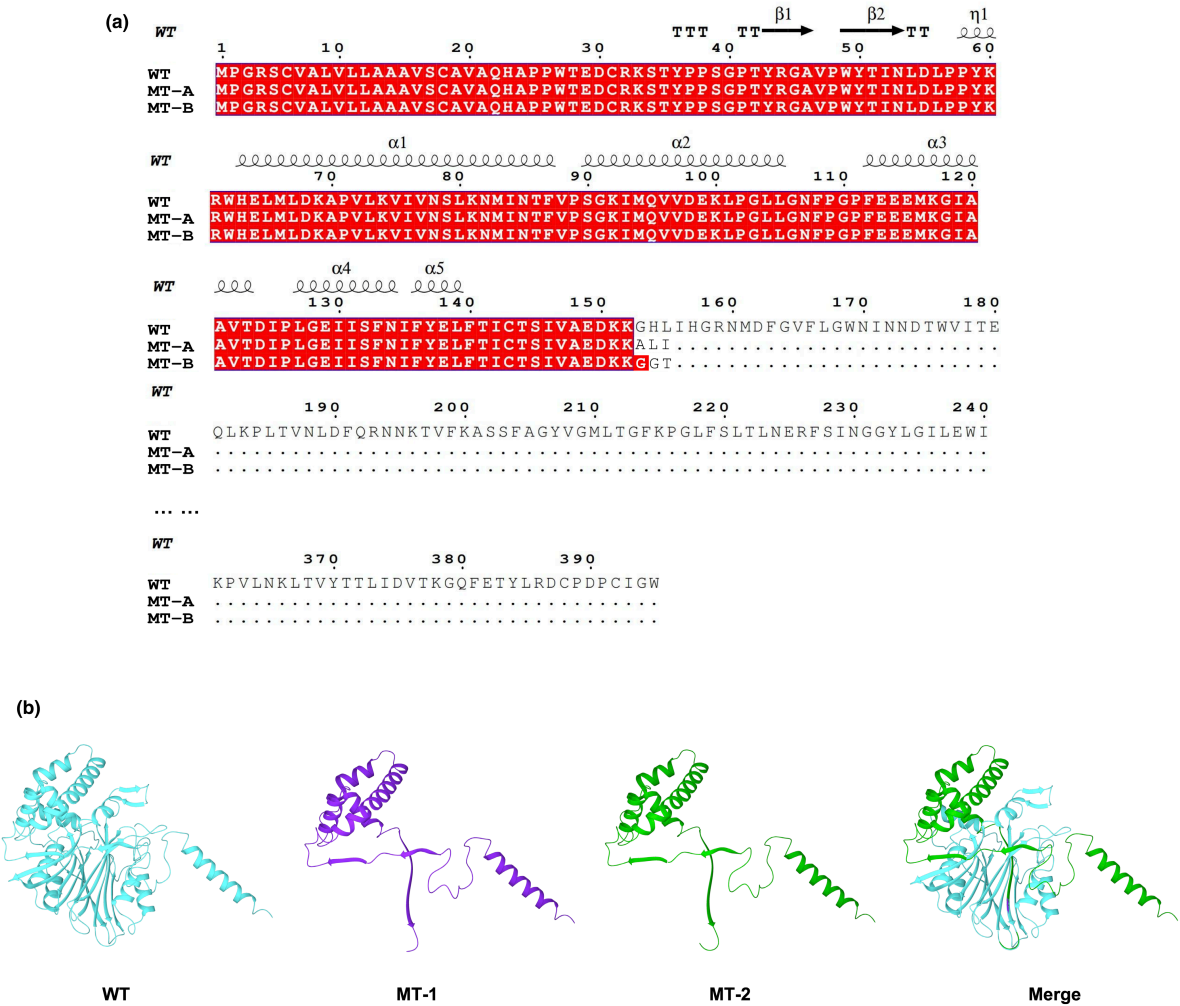
The *ASAH1* c.458‐2A>T variant results in premature stop codons and truncated proteins. (a) The translational process of WT, MT‐A, and MT‐B, both causing a premature stop codon at position 155. (b) WT protein, MT‐A, and MT‐B resulting in a truncated protein, and merge of the WT protein, MT‐A, and MT‐B proteins.

To explore whether the 56 bp insertion or exon 7 skipping was associated with downstream *ASAH1* dysregulation, we analyzed the coding potential of their sequences. The variation was first aligned back into the WT allele and deciphered by the central dogma. However, translation of the nucleotide to protein sequence revealed that not only did the 56 bp insertion induce a coding‐frame shift leading to premature translational termination, but also the exon 7 deletion incurred stop codons that interrupted the translational process and resulted in truncated proteins (Figure 4).

## 4 DISCUSSION

Here, we describe a case of *ASAH1*‐associated disease that presented as hydrops in the first trimester. The fetus was extraordinary because of its severe generalized edema with NT 6.7 mm at 13 weeks of gestation. After 5 days, the fetus died in utero because of continued progress. After excluding thalassemia carriers from the parents, we subjected the fetus and parents to CNV‐Seq and WES, respectively, and detected a novel splice site variant in *ASAH1*. This has not been previously reported in any public database or literature. According to ACMG guidelines, c.458‐2A>T of *ASAH1* is an acceptor variant 2 bp upstream of exon 7, which causes truncation or skipping of the downstream exon. Results from minigene splicing analyses demonstrated that the c.458‐2A>T variant abolished the canonical splicing of intron 6, thereby activating two aberrant splicing products, namely 56 bp retention and exon 7 skipping. Predictions suggested that these aberrant splicing products can interrupt the translation process, resulting in truncated proteins. These findings reveal the characteristics of this novel splicing site variant in the mutational spectrum of *ASAH1* in *ASAH1*‐related diseases and its aberrant effect on splicing and are expected to aid genetic counseling and accurate prenatal diagnosis.

Previous studies have shown that variants of *ASAH1* result in a lack of ACDase activity and, in turn, cause a spectrum disorder that includes FD and SMA‐PME (Zielonka et al., 2018). Both disorders are caused by variation in the *ASAH1* sphingolipid metabolism that originates from a deficiency in lysosomal acid ceramidase, in an autosomal recessive manner. At present, only about 200 cases with variants in *ASAH1* have been identified, of which more than two thirds are associated with FD (Elsea et al., 2020; Yu et al., 2018). The most common manifestations of classical FD usually occur within 4 months of birth and include the accumulation of joint contractures, progressive hoarseness of voice, formation of subcutaneous nodules, failure to thrive, and death at around 2 years of age. The clinical presentation of SMA‐PME is variable and is characterized by childhood‐onset progressive muscle weakness and seizures (Kim et al., 2016).

In a mouse model, consecutive deposition of ceramide in tissues leads to multiple organ system pathologies, with potential impacts on the spleen, bone, central nervous system, cartilage, immune system, lungs, and other internal organs (Alayoubi et al., 2013). The diagnosis of *ASAH1*‐related disorders is challenging mainly because of the complexity and variations in clinical symptoms. Patients may develop severe pathologies that lead to death during infancy or cardinal symptoms may take years to emerge, during which they are often initially misdiagnosed or not diagnosed until adulthood (Bonafé et al., 2016; Hugle et al., 2014). Ceramide, the main lipid accumulation in *ASAH1*‐related disorders, was not identified until 1967, while ceramide metabolism disorder was caused by pathogenic variants of *ASAH1* in 1996 (Koch et al., 1996; Prensky et al., 1967). Exome sequencing has been widely applied, either alone or in combination with biochemical assays, to provide a conclusive diagnosis of ACDase deficiency. These results provide a better understanding of the pathogenesis, diagnosis, and treatment of individuals with *ASAH1*‐related disorders (Gan et al., 2015; Teoh et al., 2016). Owing to scarce resources, the primary methods for diagnosing *ASAH1*‐related disorders in underdeveloped nations are clinical signs, enzyme activity measurements, and histological diagnoses (Schäfer et al., 1996; Van Lijnschoten et al., 2000). Although we did not perform an acid ceramidase enzyme activity assessment on the patient in this study who presented with severe hydrops fetalis at 13 weeks of gestation, intrauterine symptoms, *ASAH1* sequencing, and coding potential prediction results strongly supported *ASAH1*‐related disorder diagnosis.

*ASAH1* is relatively small, comprising 14 exons that range between 46 and 1200 bp long (Schuchman et al., 2015). According to the NCBI ClinVar public archive, which contains deletion/duplication, CNVs and single nucleotide variants, 105 pathogenetic variations, and 48 likely pathogenic variations have been identified throughout *ASAH1*. Most of these variations appear to be missense mutations. Approximately, 10%–15% of these variants comprise different variations of splicing errors, most of which affect canonical splice sites at the exon/intron boundaries and consequently lead to loss of protein function (Farber, 1952; Koch et al., 1996; Su et al., 2021). The ACDase was composed of an α (13 kDa) and a β (40 kDa) subunit, and the splice site c.458‐2A>T that we reported was located in the region that encodes the β‐subunit. (Alves et al., 2013; Bashyam et al., 2014; Elsea et al., 2020).

Little is known about the overall dynamics of *ASAH1*‐related disorder prevalence, mainly due to the extreme rarity of conditions (Yu et al., 2018). Notably, patients with mild or intermediate symptoms tend to live longer, with the oldest patient reported to be over 60 years of age. In more serious FD cases, the shortest‐lived patients present with hydrops (Solyom et al., 2017). Of the two cases who presented with hydrops fetus, one was a 29‐week‐stillborn fetus with mild internal hydrops, foamy cells, and well‐preserved spleen (Van Lijnschoten et al., 2000). The other case was a female preterm infant, 29 weeks of gestation, with severe hydrops fetalis who died 3 days postnatum due to disseminated intravascular coagulation. Autopsy findings revealed an enlarged abdomen filled with hemorrhagic ascites, hepatosplenomegaly, and many white granulomas on the peritoneal surfaces of the liver, spleen, and lungs (Kattner et al., 1997; Schäfer et al., 1996). Genetic analysis revealed that the parents were *ASAH1* mutations carrier (Bashyam et al., 2014). In addition to severe hydrops fetalis, cardiac abnormalities were observed by ultrasound in our report. We speculate that abnormalities of the heart may be related to increased nuchal translucency (NT). Studies have reported that increased NT is likely to result from abnormal lymphatic development and is strongly associated with cardiac defects (Burger et al., 2015; Minnella et al., 2020). However, diverse clinical manifestations and progressive courses suggest that there maybe more variable forms of *ASAH1*‐related disorders and that more careful clinical investigation is warranted.

Previous studies have shown that mice homozygous for the complete loss‐of‐function *ASAH1* allele undergo apoptotic death at the two‐cell stage due to the accumulation of ceramide. These results indicate that ACDase expressed in human cumulus cells and follicular fluid, which are essential components of the environment and the level of expression is positively correlated with the quality of human embryos formed in vitro (Eliyahu et al., 2007; Eliyahu et al., 2010). Our findings are in line with the above studies, which indicate that ACDase is essential for oocyte and embryo survival, while complete loss‐of‐function variation in FD individuals may lead to early embryonic lethality. Importantly, ACDase is essential not only during early embryonic development but also during postnatal life. Previous studies have shown that suppression of ACDase activity contributes to lipid storage disease, thereby inducing default apoptosis in cells (Eliyahu et al., 2007). As previously reported in two cases of fetal death, lipid storage disorders are fatal during embryonic development. Studies have shown that surviving FD patients exhibit missense mutations that may be partly function preserved in *ASAH1*, rather than large fragment deletions, insertions, nonsense mutations, or frameshift mutations that cause complete loss‐of‐function (Bao et al., 2020; Elsea et al., 2020). Herein, we demonstrate that the acceptor splice site variant (c.458‐2A>T) in intron 6 of *ASAH1* can cause truncation or exon skipping, which is typically accompanied by loss‐of‐function subsequently leading to severe hydrops fetalis and early embryonic lethality.

In recent decades, extensive progress has been made about *ASAH1*‐related disorders diagnosis and treatment. Studies have shown that physical and anti‐inflammatory therapies can be used to manage pain and mobility issues, while surgical intervention can eliminate nodules in the hands and oral cavity when necessary (Mitchell et al., 2016; Moritomo et al., 2002). Another treatment option, hematopoietic stem cell transplantation (HSCT), has been shown to significantly alleviate pain and improve mobility in FD patients without CNS involvement (Ehlert et al., 2007). Remarkably, many other ongoing studies aimed to increase ACDase activity and decrease ceramide levels, as gene therapies using lentiviral or retroviral vectors and enzyme replacement therapy (ERT) with recombinant human ACDase, and are expected to improve life expectancy in FD patients in the future (Pewzner‐Jung et al., 2014; Schuchman et al., 2015). Gene therapies for monogenic lysosomal storage disorders are currently being tested in clinical trials (Huang et al., 2017; Tardieu et al., 2017).

Definitive diagnosis is very important since the variation in *ASAH1* causes a spectrum disorder that includes FD and SMA‐PME (Genovese et al., 2016). A broad age range at onset coupled with rapid disease progression may also be quite variable, even among individual patients. Exome sequencing, in combination with biochemical assays, is particularly informative for patients with nonclassical or mild symptoms (Gan et al., 2015; Zielonka et al., 2018). In the absence of biochemical assays, the symptoms, definitive genetic diagnosis, and in silico prediction can be particularly significant for the diagnosis of related disorders, thus aiding genetic counseling for couples who prepare for pregnancy and prenatal diagnosis for the fetuses suspected to be inherited in utero.

## 5 CONCLUSION

Here, we present a case of *ASAH1*‐associated disease presenting with severe hydrops fetalis. These findings provide new insights into additional phenotypes and a novel intronic pathogenic *ASAH1* variant present in non‐consanguineous parents that was found to have an aberrant effect on splicing. Collectively, these findings provide a better understanding of *ASAH1*‐related disorders during prenatal examination and are expected to guide accurate diagnosis as well as genetic counseling for the family, which effectively blocks the transmission of genetic birth defects. Improved understanding of the disease will not only enable the identification and accurate diagnosis of more patients, but also effective management of the group.

## AUTHOR CONTRIBUTIONS

Shujuan Yan and Can Liao designed this project. Fu Fang and Hang Zhou organized the genetic analysis and bioinformatic analysis. Ruibin Huang and You Wang collected the clinic data. All authors have read and approved the final article.

## FUNDING INFORMATION

This work was supported by the Subproject of the National Key R&D Program (2021YFC2701002), the National Natural Science Foundation of China (82060388, 81801461, 81873836, 81771594, 81671474, and 81501267), and the Guizhou Science and Technology Department (QKHJC[2020]1Y424).

## CONFLICT OF INTEREST STATEMENT

The authors declare that they have no known competing financial interests or personal relationships that could have influenced the work reported in this study.

## ETHICS STATEMENT

This study was approved by the Ethics Committee of Guangzhou Women and Children's Medical Center.

## Data Availability

The data that support the finding of this study are available from the corresponding author upon reasonable request.
